# Multidisciplinary intensive education in the hospital improves outcomes for hospitalized heart failure patients in a Japanese rural setting

**DOI:** 10.1186/1472-6963-14-351

**Published:** 2014-08-19

**Authors:** Yoshiharu Kinugasa, Masahiko Kato, Shinobu Sugihara, Kiyotaka Yanagihara, Kensaku Yamada, Masayuki Hirai, Kazuhiro Yamamoto

**Affiliations:** Division of Cardiovascular Medicine, Department of Molecular Medicine and Therapeutics, Faculty of Medicine, Tottori University, 36-1 Nishicho, 683-8504 Yonago, Japan

**Keywords:** Multidisciplinary intervention, Heart failure management program, Team education, Self-care behavior

## Abstract

**Background:**

Heart failure (HF) patients living in rural areas have a lack of HF knowledge and poor self-care because of limited medical care access. Multidisciplinary education to improve self-care behavior is indispensable for such patients. The present study evaluated whether intensive inpatient education improved outcomes of hospitalized HF patients in a Japanese rural setting.

**Methods:**

An inpatient HF management program based on multidisciplinary team intervention was applied to hospitalized HF patients in a Japanese rural area. We defined patients treated within the program from May 2009 to April 2011 as the intervention group (n = 144), and those treated with the usual care from May 2006 to April 2009 as the usual care group (n = 133). The composite endpoints of HF hospitalization and all-cause mortality were compared between the two groups.

**Results:**

Compared with patients in the usual care group, those in the intervention group more often received the optimal interventions such as discharge use of β-blockers, cardiac rehabilitation, pre-discharge diagnostic tests, and multidisciplinary intensive education including nurse-led patient education, pharmacist’s medication teaching, and dietitian’s nutritional guidance (all P < 0.05). The incidence of the composite endpoints significantly decreased after introducing the program (P < 0.001). Among a number of interventions, multidisciplinary intensive education was the most effective intervention to improve the primary outcome (P < 0.001).

**Conclusions:**

Multidisciplinary intensive education is a key strategy for helping improve the outcome for Japanese HF patients in a rural setting. Our data may give a positive impact on the improvement of healthcare system in Japan.

## Background

HF has become a leading cause of hospitalization in adults older than 65 years, and its increased prevalence imposes a burden on the healthcare system [1]. Despite a number of pharmacological HF treatments shown to improve outcomes, the prognosis of these patients remains poor [2]. Furthermore, patients with HF have a high rate of post-discharge re-hospitalization with episodes of acute deterioration throughout their lifetime, which may lead to their gradual functional decline and poor prognosis [3]. Therefore, the development of strategies for preventing HF readmission is necessary to raise quality of life (QOL) and improve prognosis in these patients [4, 5].

HF patients living in rural areas are more likely to be readmitted than those living in urban areas [6]. They lack HF knowledge and have poor self-care because of limited medical care access, indicating that a multidisciplinary approach to improving self-care behaviors may be more important for HF patients in rural areas than those in urban areas [7–9]. A multidisciplinary HF management program, including optimal medications, comprehensive HF education, and specialized follow-up including telephone contact and home visits by a multidisciplinary HF team, has been shown to improve clinical outcomes in patients with HF [4, 5, 10–14]. Although such strategies have been established in Europe and the United States, no established HF management program is available in Japan [14]. In addition, these resource-intensive outpatient programs are mainly limited to major urban medical centers and are generally unavailable in rural or limited medical care access areas [7].

We tested the hypothesis that multidisciplinary inpatient education provides considerable knowledge about HF and optimal self-care management, and improves post-discharge outcomes of hospitalized HF patients in a Japanese rural area with limited access to medical care in outpatient settings.

## Methods

### Subjects

The present study enrolled 277 consecutive patients hospitalized in Tottori University Hospital with a primary diagnosis of HF from May 2006 to April 2011. Our hospital is the core hospital in Tottori prefecture, a rural area with the lowest total population of all prefectures in Japan. There are nine hospitals (more than 200 beds) with divisions of cardiovascular medicine in this area, and none of them has a cardiac care unit. In addition, only 57 cardiovascular specialists work in the Tottori prefecture, compared with 1833 in the Tokyo metropolitan area. Thus, Tottori is one of the rural areas with limited access to cardiovascular health resources.

HF was defined according to Framingham criteria, together with pulmonary congestion on X-rays or pulmonary hypertension evaluated by Doppler echocardiography [15]. Acute coronary syndrome was excluded. Patients with the following criteria were also excluded: severe valve disease, congenital disease, complete atrioventricular block, sick sinus syndrome, pericardial disease, primary pulmonary hypertension, and pulmonary artery embolism. Patients who died in hospital were also excluded. All subjects were treated with diuretics, vasodilators, and/or inotropes for symptom relief after admission.

We have developed and applied a multidisciplinary HF management program for patients hospitalized with HF since May 2009. The subjects were divided into two groups according to whether they were admitted before or after we introduced the program. The intervention group consisted of 144 consecutive patients who were admitted from May 2009 to April 2011 and treated according to the multidisciplinary HF management program, while the usual care group (historical control group) consisted of 133 consecutive patients who were admitted from May 2006 to April 2009 and not treated according to the program. Clinical events after discharge were compared between the two groups.

### Multidisciplinary HF management program

The multidisciplinary HF management program focused on the comprehensive medical and non-medical interventions directed by the multidisciplinary HF team during hospital admission. The HF team consists of an experienced HF cardiologist, a cardiovascular nurse, pharmacist, dietitian, physical therapist, sonographers, and a social worker. Components of the program are as follows.

1. Optimization of HF therapy by HF cardiologist
  Experienced HF cardiologists check the medical record, and recommend the evidence-based pharmacological/non-pharmacological therapies to the attending physicians.
2. Cardiac rehabilitation by physical therapist
  Cardiac rehabilitation is applied for all patients without any contraindication to prevent bed-rest deconditioning during the hospital stay. Physical therapists also educate patients about optimal daily activity to avoid being overworked after discharge.
3. Multidisciplinary team education by nurse, pharmacist, and dietitian
  Multidisciplinary team education consists of the three types of education: nurse-led patient education, pharmacist’s medication teaching, and dietitian’s nutritional guidance. Experienced cardiovascular nurses educate patients and their caregivers about symptom monitoring and self-care management using a teaching booklet. Daily weight recording is recommended for all patients, and in the case of a sudden unexpected weight gain, consultation with attending physicians is recommended. Pharmacists educate patients about the effects and side effects of each drug. They also check medication adherence before admission, and provide a plan to improve medication adherence. Dietitians assess the nutritional status in each patient, and recommend appropriate daily energy intake, and avoidance of excessive salt and fluid intake.
4. Team conference by multidisciplinary HF team
  A team conference was scheduled every week and each patient’s issues associated with worsening HF were discussed.
5. Pre-discharge assessment of congestion
  To avoid incomplete relief from fluid overload, pre-discharge echocardiography was conducted by experienced sonographers, and plasma B-type natriuretic peptide (BNP) level was measured 1 week before discharge. If BNP level and/or echocardiography indicated incomplete relief from congestion, discharge was postponed and further treatment was added.
6. Discharge care planning
  In patients with socio-environmental issues, social workers facilitated care planning, and provided a social care service. Follow-up with the cardiovascular specialists after discharge was also recommended for patients with severe HF and a history of repeated HF hospitalization.

### Data collection

Medical records were retrospectively reviewed with regard to demography, medical history, comorbidities, laboratory data, echocardiograms, medications, and clinical course. Left ventricular ejection fraction (LVEF) and estimated glomerular filtration rate were calculated as previously described [15, 16]. The study subjects were followed for 1 year, and follow-up data were obtained from medical records or telephone interviews. We evaluated the composite endpoints of all-cause mortality and unplanned HF re-hospitalization during the follow-up period.

This retrospective study conforms to the principles outlined in the Declaration of Helsinki and the guiding principles for epidemiologic studies of the Ministry of Health, Labour and Welfare, Japan, and was approved by the research ethical committees of Tottori University. Following the guiding principles for epidemiologic studies of the Ministry of Health, Labour and Welfare, Japan, research information about this study was released to the public, and we were allowed to obtain and analyze the data without obtaining informed consent from each patient.

### Statistical analysis

**Figure 1:**
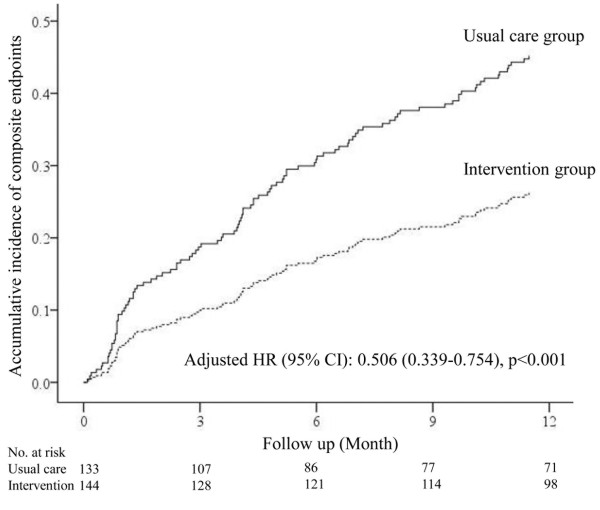
**Cumulative event curves for the composite end points for patients in the usual care and intervention groups.** Cumulative event curves were adjusted for age and sex. HR: Hazard ratio. CI: Confidence interval.

**Figure 2:**
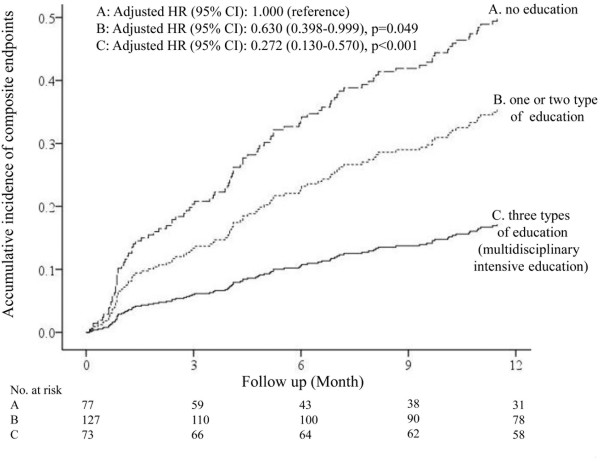
**Adjusted cumulative event curves for the composite end points among three groups according to the number of education interventions received.** Cumulative event curves were adjusted for age, sex, left ventricular ejection fraction, discharge use of β-blockers, discharge use of angiotensin-converting enzyme inhibitors, cardiac rehabilitation, pre-discharge assessment of echocardiography/B-type natriuretic peptide level, and follow-up with the cardiologists. HR: hazard ratio. CI: Confidence interval.

**Figure 3:**
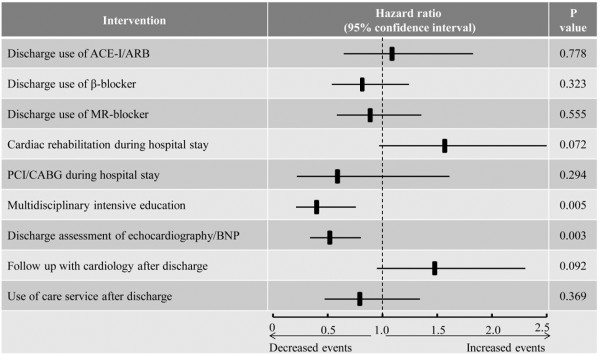
**The effect of each medical and non-medical intervention on the primary outcome.** Cox hazard model was adjusted for age and sex. ACE-I: Angiotensin-converting enzyme inhibitor. ARB: Angiotensin receptor blocker. MR: Mineralocorticoid receptor. PCI: Percutaneous coronary intervention. CABG: Coronary artery bypass graft. BNP: B-type natriuretic peptide.

**Figure 4:**
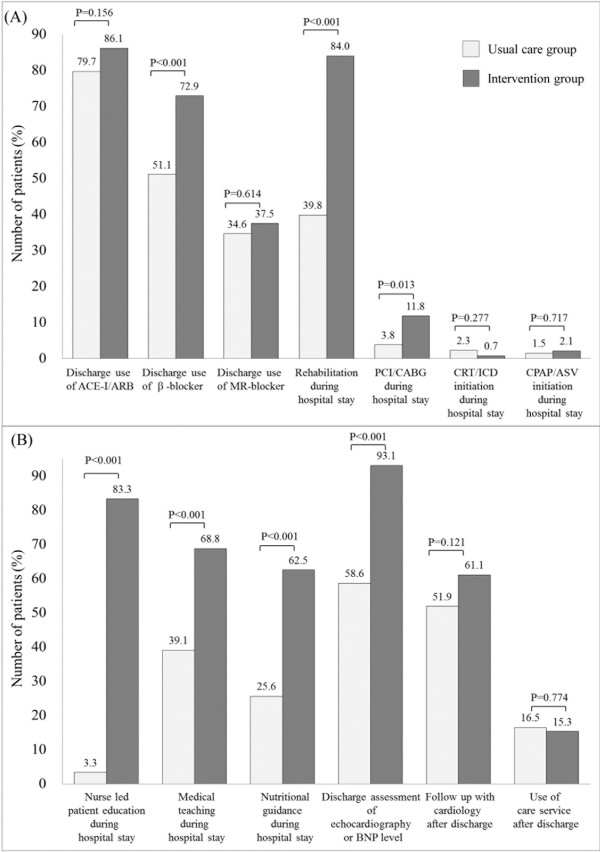
**In-hospital management in the usual care and intervention groups, including pharmacological and non-pharmacological intervention (A), and educational intervention and discharge assessment and planning (B).** ACE-I, angiotensin-converting enzyme inhibitor; ARB, angiotensin receptor blocker; MR, mineralocorticoid receptor; PCI, percutaneous coronary intervention; CABG, coronary artery bypass grafting; CRT, cardiac resynchronization therapy; ICD, implantable cardiac defibrillator; CPAP, continuous positive airway pressure; ASV, adaptive servo ventilation. BNP: B-type natriuretic peptide. *care service including nurse home visit or day care service.

Continuous variables are expressed as mean ± standard deviation, and categorical variables are expressed as percentages. Differences in continuous variables were compared using the t-test for two groups, and analysis of variance for multiple groups. Categorical variables were compared using the χ^2^ test. The Cox proportional hazards models were used to assess the effect of interventions on the primary outcome (Figures 1, 2 and 3). Cumulative event curves as shown in Figures 1 and 2 were adjusted for age, sex, and the different baseline characteristics among each group (P < 0.05). The Cox hazard model as shown in Figure 3 was adjusted for age, sex, and all of the interventions as shown in Figure 4. However, device therapies were not considered for the analysis because of their low prevalence. A P value < 0.05 was considered statistically significant. All analyses were performed using IBM SPSS Statistics version 20 (SPSS, Inc, Chicago, IL, USA).

## Results

### Baseline patient characteristics

Baseline patient characteristics at discharge in the usual care and intervention groups are shown in Table 1. The mean age of the overall cohort was 74 ± 13 years and 59.6% were male. The prevalence of ischemic heart disease was 36.5%. Previous history of HF hospitalization was found in 22.4% of patients. The mean LVEF was 45.4 ± 16.0%. The two groups had similar baseline characteristics including age, sex, HF etiology, LVEF, New York Heart Association (NYHA) class, and other parameters as shown in Table 1. Available data of echocardiographic index of congestion (vena cava diameters, tricuspid regurgitation peak gradient, and left atrial diameter) at discharge were also similar between the two groups (data not shown).

**Table 1:** **Baseline characteristics at discharge between the control and usual care groups**

	Overall (n = 277)	Usual care (n = 133)	Intervention (n = 144)	P value
Age (years)	74 ± 13	74 ± 13	75 ± 13	0.657
Male (%)	59.6	58.6	60.4	0.764
Ischemic heart disease (%)	36.5	33.1	39.6	0.261
NYHA class III/IV (%)	10.5	9.0	11.8	0.450
Prior HF admission (%)	22.4	21.1	23.6	0.610
LVEF (%)	45.4 ± 16.0	44.9 ± 16.5	45.9 ± 16.2	0.624
HFpEF (LVEF ≥ 45 %) (%)	46.6	42.9	50.0	0.234
Vital sign				
SBP (mmHg)	118 ± 22	117 ± 23	119 ± 21	0.586
Heart rate (beats/min)	68 ± 12	69 ± 13	68 ± 10	0.623
Comorbidity condition				
Hypertension (%)	58.5	52.6	63.9	0.057
Atrial fibrillation (%)	41.9	44.4	39.6	0.421
Diabetes (%)	37.5	38.3	36.8	0.784
Dyslipidemia (%)	27.1	26.3	27.8	0.784
COPD (%)	9.0	9.0	9.0	0.999
Laboratory values				
Hemoglobin (g/dl)	11.7 ± 2.3	11.8 ± 2.3	11.6 ± 2.4	0.482
Sodium (mEq/dl)	137.8 ± 4.0	138.0 ± 4.2	137.6 ± 3.8	0.394
BUN (mg/dl)	34.1 ± 20.6	33.4 ± 21.1	34.8 ± 20.1	0.577
Creatinine (mg/dl)	1.49 ± 1.19	1.48 ± 1.34	1.50 ± 1.04	0.874
eGFR (ml/ml/1.73 m^2^)	48.3 ± 29.1	49.2 ± 27.4	47.5 ± 30.7	0.631
BNP (pg/ml)*	325 ± 395	360 ± 435	306 ± 372	0.366

Data are mean ± standard deviation.

HF: Heart failure. NYHA: New York Heart Association. SBP: Systolic blood pressure. LVEF: Left ventricular ejection fraction. HFpEF: Heart failure with preserved EF. COPD: Chronic obstructive pulmonary disease. BUN: Blood urea nitrogen. eGFR: estimated glomerular filtration rate. BNP: B-type natriuretic peptide.

*available data for 191 subjects.

### The effect of the multidisciplinary HF management program on in-hospital management

**Figure 5:**
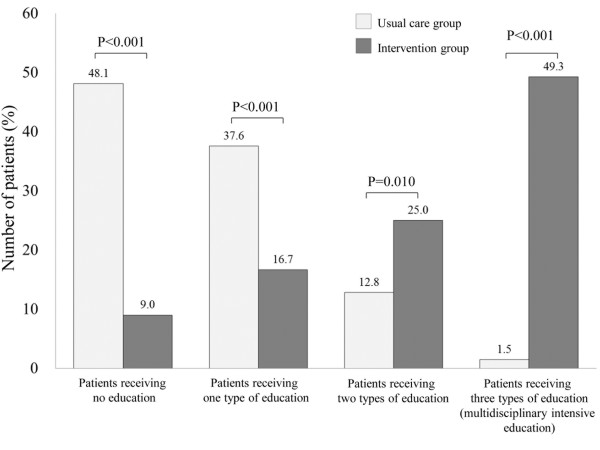
**The distribution of education types received by patients in the usual care and intervention groups.**

Medical and non-medical interventions between the usual care and the intervention groups are shown in Figure 4A and B. Patients in the intervention group received the optimal medical treatments such as discharge use of β-blockers, cardiac rehabilitation, and coronary artery revascularization more often than those in the usual care group (all P < 0.05, Figure 4A). They were also more likely to receive educational interventions such as nurse-led patient education, pharmacist’s medical teaching, and dietitian’s nutritional guidance compared with those in the usual care group (all P < 0.01, Figure 4B). Furthermore, patients in the intervention group more often received multiple (two or three types of) education compared with those in the usual care group (all P < 0.05, Figure 5). Diagnostic tests such as echocardiography or BNP level were more likely to be conducted before discharge in patients in the intervention group compared with those in the control group (P < 0.01, Figure 4B). The median (interquartile range) length of hospital stay in the intervention group was significantly longer than that in the usual care group: 27 (19–38) days versus 22 (16–38) days (P = 0.023).

### Effect of the multidisciplinary HF management program on clinical outcomes

Composite endpoints of all-cause mortality and HF hospitalization occurred in 61 patients (45.9%) in the usual care group, compared with 40 patients (27.8%) in the intervention group. The specific distribution of the composite endpoints was as follows: all-cause mortality: 12 patients (9.0%) in the usual care group and 17 patients (11.8%) in the intervention group; HF hospitalization: 49 patients (36.8%) in the usual care group and 23 patients (16.0%) in the intervention group. Cox hazard analysis showed that patients in the intervention group had approximately 50% reduction of risk of the composite endpoints compared with those in the usual care group: hazard ratio (HR) 0.506; 95% CI: 0.339–0.754; P < 0.001 (Figure 1).

### The effect of education of patients on clinical outcomes

To assess the effects of education of patients on the primary outcome, study subjects were divided into three groups according to the number of the education types patients received: group A: no education; group B: one or two types of education; and group C: three types of education (multidisciplinary intensive education). As shown in Figure 2, the implementation of a greater number of education types was associated with a decreased risk for the primary endpoint: HR 0.630; 95% CI: 0.398-0.999; p=0.049 (group B versus group A), HR 0.272; 95% CI: 0.130-0.570; p<0.001 (group C versus group A). Cox hazard analysis identified multidisciplinary intensive education as the most effective intervention to reduce the risk of the primary endpoint among all medical and non-medical interventions: HR 0.387; 95% CI: 0.200–0.738; P < 0.001 (Figure 3).

## Discussion

The present study shows that a multidisciplinary HF management program during hospitalization improved quality of care with regard to the optimal medical treatment, comprehensive team education, and pre-discharge diagnostic tests and improved post-discharge outcome of Japanese HF patients living in rural areas. Furthermore, among a number of interventions, multidisciplinary intensive education by a nurse, pharmacist, and dietitian was the most effective intervention to reduce the risk of the primary outcome. These results suggest that a multidisciplinary educational approach is a key strategy for helping prevent re-hospitalization for HF in Japanese HF patients in a rural setting.

HF patients living in rural areas are at high risk of HF readmission [6]. They lack knowledge about HF, and their self-care is poor because of limited medical care access. Therefore, effective educational interventions to improve self-care behaviors are needed more for HF patients in rural areas than those in urban areas [7–9]. However, most previous HF management programs focused on outpatient-based educational interventions [4, 5, 11–14], and these resource-intensive outpatient programs are generally unavailable in rural areas with limited medical care access. In this study, the multidisciplinary HF management program during hospitalization resulted in an approximately 50% risk reduction for the primary outcome in HF patients in a rural area of Japan. This result is close to the effects of post-discharge intervention found in western countries and Japan [11–14]. Reduction in re-hospitalization may reduce clinical costs: HF is the most frequent medical reason for re-hospitalization within 30 days [17]. The current finding may well contribute to reducing the socioeconomic burden of HF.

The present study, for the first time, demonstrated that intensive education conducted by a nurse, pharmacist, and dietitian particularly played an important role in improving the post-discharge outcome of Japanese HF patients in a rural setting, even when conducted only during hospitalization (Figure 3). Thus, educational intervention, even for a brief period, has a beneficial effect on the clinical outcome of these patients. Importantly, we also showed that implementation of an increased number of education types was associated with a decreased risk for the primary endpoints (Figure 2). Nurse-led patient education is an indispensable component of a disease management program. The current results show that a pharmacist’s and/or dietitian’s intervention during hospitalization provides additive beneficial effects, which is partly compatible with previous studies assessing the effects of outpatient interventions of pharmacists or dietitians [18, 19]. Multiple, not single, intensive education types may improve the overall quality of education, patients’ self-care behavior, and adherence to the optimal treatment in Japanese HF patients in rural settings.

In addition to educational interventions, pre-discharge diagnostic tests were also independently associated with decreased risk for the primary endpoints: HR 0.511; 95% CI: 0.329–0.794 (Figure 3). Incomplete relief from fluid overload is one cause of early readmission [20], and the appropriate pre-discharge assessment of congestion and further treatment may prevent incomplete treatments in the hospital [21].

Although most studies reported favorable effects of HF management programs, some reports have shown negative findings [22–24]. The Coordinating Study Evaluating Outcomes of Advising and Counseling in Heart Failure (COACH) was one of the largest multicenter, randomized, controlled trials for evaluating the effect of a nurse-led disease management program on outcomes in patients with HF [23]. This study showed that nurse-led intensive support did not reduce the combined endpoints of death and HF hospitalization. There are several explanations for the discrepancies between our study and the COACH study. Patients in the COACH study were managed adequately already [23], whereas the usual care group of our study living in rural areas might be less likely to receive optimal medications and education because they had limited medical care access before admission. Thus, our interventions were more likely to show additional benefits. In addition, our study cohort had a lower prevalence of severe HF (NYHA III/IV) than the COACH study cohort. This may partly be explained by the difference in medical care systems between western countries and Japan. In Japan, longer hospitalization is allowed, and HF symptoms are better controlled at discharge. These data will contribute to the discussion of the optimal design of disease management programs to fit patients and health care systems based on the situation in each country [23].

It is also important to coordinate regional collaboration with primary physician and care services in rural settings [21, 25, 26]. Our program recommended follow-up with cardiovascular specialists after discharge. However, there was no significant increase in the rate of this follow-up after the introduction of our program because of the limited cardiovascular health resources in this rural setting. Therefore, collaborative care with a primary physician (non-cardiologist) is required particularly for HF patients in rural areas [25, 26]. Social support is also important for such patients [27]. We found that the use of a care service after discharge (nurse home visit or day care service) tended to reduce the incidence of the primary outcome (HR 0.783; 95% CI: 0.460–1.335; Figure 3). HF patients living in rural areas are older and have several socio-environmental issues, meaning social support is more beneficial for these patients [7–9]. Further investigations are necessary to evaluate the beneficial effect of regional collaborative care for Japanese patients with HF in rural areas.

Our study has several limitations. It was not randomized, but consisted of a “before and after” single-center design. General standards of care during hospitalization and after discharge may have improved in the time between our control and intervention periods, and this may have affected clinical outcomes in the intervention group. Second, previous studies reported the beneficial effects of disease management programs on patients’ QOL and psychological status [14]. However, we could not evaluate such outcomes. In addition, we could not evaluate cognitive function, which may affect the impact of educational intervention on clinical outcomes. Third, we did not analyze medical care costs. Patient education and rehabilitation may have prolonged hospital stay and increased clinical cost in the intervention group. However, repeated HF hospitalization also increases health care costs, and the cost savings generated by multidisciplinary management programs may potentially offset these additive clinical costs [4]. Recently, the law in the United States has required the Centers for Medicare and Medicaid Services to reduce payments to hospitals with excess re-hospitalization within 30 days of a discharge from HF, myocardial infarction, and pneumonia [28]. Among the three conditions, HF has the highest 30-day re-hospitalization rate [17]. Thus, even in western countries, the beneficial effects of pre-discharge education may outweigh its additive cost for this education or the associated prolonged hospital stay. Further well-designed, large, prospective, randomized trials are required to confirm the effects of multidisciplinary team interventions during hospitalization on various clinical outcomes in Japanese patients with HF in rural areas.

## Conclusions

An inpatient HF management program improved quality of care with regard to the optimal medical treatment, comprehensive team education, and pre-discharge diagnostic tests and improved post-discharge outcome of Japanese HF patients living in rural areas. Furthermore, among a number of interventions, multidisciplinary intensive education was the most effective intervention to reduce the risk of the primary outcome, indicating that a multidisciplinary educational approach is a key strategy for helping improve the outcome for these patients. Our data may give a positive impact on the improvement of healthcare system in Japan.

## Abbreviations

HF: Heart failure
QOL: Quality of life
LVEF: Left ventricular ejection fraction
NYHA: Newyork heart association
HR: Hazard ratio
CI: Confidence interval.
